# Inhalation Exposure of Organophosphate Pesticides by Vegetable Growers in the Bang-Rieng Subdistrict in Thailand

**DOI:** 10.1155/2009/452373

**Published:** 2010-02-07

**Authors:** Somsiri Jaipieam, Parichart Visuthismajarn, Wattasit Siriwong, Marija Borjan, Mark G. Robson

**Affiliations:** ^1^National Center of Excellence for Environmental and Hazardous Waste Management (NCE-EHWM), Chulalongkorn University, Bangkok 10330, Thailand; ^2^Faculty of Environmental Management, Prince of Songkla University, Songkhla 90112, Thailand; ^3^College of Public Health Sciences, Chulalongkorn University, Bangkok 10330, Thailand; ^4^New Jersey Agricultural Experiment Station, Rutgers University, New Brunswick, NJ 08901, USA

## Abstract

This study investigated inhalation exposure to organophosphate pesticides (OPPs) and evaluated the associated health risks to vegetable growers living in the Bang-Rieng agricultural community. Air samples were collected by using personal sampling pumps with sorbent tubes placed in the vegetable growers' breathing zone. Samples were collected during both wet and dry seasons. Residues of organophosphate pesticides, that is, chlorpyrifos, dicrotofos, and profenofos, were analyzed from 33 vegetable growers and 17 reference subjects. Results showed that median concentrations of OPPs in air in farm areas were in the range of 0.022–0.056 mg/m^3^ and air in nonfarm areas in the range of <0.0016–<0.005 mg/m^3^. The concentration of the three pesticides in the vegetable growers was significantly higher than that of the references during both seasons. The results also indicate that the vegetable growers may be at risk for acute adverse effects via the inhalation of chlorpyrifos and dicrotofos during pesticide application, mixing, loading, and spraying. It is suggested that authorities and the community should implement appropriate strategies concerning risk reduction and risk management.

## 1. Introduction

Thailand exports a large amount of agricultural products to the world market every year. The agricultural labor force makes up about 55.7% of the Thai population. Most of this workforce's income is generated through sales of farm products such as rice, fruit, cassava, and para-rubber [1]. To minimize crop damage and to increase land productivity, the use of pesticides has become more essential. The import of pesticides has increased considerably from approximately 4,000 tons in 1962 to 37,039 tons in 2001. The burden of pesticides, as applied for crop protection, may affect environmental quality and human health [2]. 

Due to the ease of purchase and the high effectiveness in pest control, farmers are applying pesticides in large quantities. Since the ban of organochlorine pesticides in Thailand, the most widely used pesticide has become organophosphate pesticides (OPPs). OPPs are very effective in the eradication of insects and they are not as persistent in the environment as organochlorine pesticides. However, OPPs can cause acute adverse human health effects. At certain doses, OPPs have adverse effects on insects and humans. OPPs inhibit acetylcholinesterase enzymes (AChEs) at nerve ending that result in a build up of acetylcholine (Ach). Ach is an important neurotransmitter. When it accumulates at a synapse due to jamming of information the passing of messages between nerve cells is prevented. Adverse effects from OPP exposure include pulmonary edema, cyanosis, muscle spasms, muscle weakness, blurred vision, respiratory difficulty and possibly death due to respiratory failure [3]. The severity of the health effects varies with exposure dose and duration.

The Bang-Rieng subdistrict is a large agricultural area in Songkhla Province, Southern Thailand. The farmers in this area grow a variety of vegetables. The Bang-Rieng community can be divided into two regions based on the patterns of agricultural practices: intensive and integrated pest management (IPM). Intensive agriculture refers to a commercial agriculture system that relies on a large market. As part of intensive practices, farmers mainly use pesticides for pest control. IPM, on the other hand, focuses on reducing pesticide use through alternative techniques such as biological control, crop rotation, or netted crop growing. 

This study focuses on intensive agricultural areas due to intensive pesticide use in Thailand. The intensive agricultural region within the Bang Reing community consists of approximately 891 rai (143 km^2^) of vegetable farm area, including 92 households (information obtained from the GIS survey by the Faculty of Natural Resources, Prince of Songkhla University, 2004). The reference areas are 7-8 km away from the farm areas, within the same subdistrict. The reference areas consist of 1,056 rai (169 km^2^) including 96 households (information obtained from database of Bang Reing subdistrict, 2004)

Farmers in the Bang-Rieng community are directly and indirectly exposed to OPP residues either by inhalation during mixing, loading, or application of pesticides; by ingestion through artesian well water; or by dermal exposure from contact with soil residues, pesticide residues on plants, or while handling pesticides. This study will focus on the inhalation route of organophosphate pesticides that is chlorpyrifos, dicrotophos, and profenofos because they are the most popular and widely used in Bang Reing agricultural area. Inhalation exposure results in the individual breathing in dilute pesticide, that is, absorbed through the surface of the lung. Chemicals then enter the blood stream and are distributed to the rest of the body [4]. 

## 2. Methods and Materials

This study was approved by the Ethical Review Committee for Research Involving Human Subjects and/or Use of Animals in Research, Health Science Group of Faculties, Colleges and Institutes, Chulalongkorn University, Thailand under document no. 097/2006. All participants signed a consent form prior to participation in this study. 

**Figure 1:**
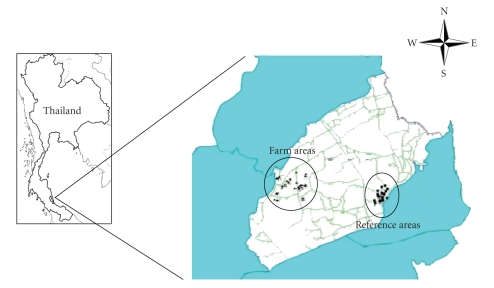
Locations of the air samples located in the Bang-Rieng subdistrict, Songkhla Province (Farm areas (▲), *n* = 33; reference areas (■), *n* = 17).

Thirty-three vegetable growers were selected as the study group. Vegetable growers were asked to participate in this study based on the farmers organophosphate pesticide use. The reference, or control, groups were made up of seventeen workers who collect latex from rubber tree plants and do not work with pesticides. The reference area was chosen based on interviews and background data. Since they live in the same subdistrict as the vegetable growers, this study will also investigate whether the reference group has inhalation exposure at work to the specific pesticides used by the vegetable growers (Figure 1). A survey was also administered by the study investigator to obtain information on subject age, body weight, and spray time.

### 2.1. Air Sampling

Altogether, 100 air samples were collected; 50 air samples were collected during the dry season (April–June 2006) and 50 during the wet season (September–October 2006). Each season, 33 air samples were collected from vegetable farm areas and 17 air samples were collected from the reference areas. Each subject provided two air samples. The air samples were treated as independent because of difference in sample collection time and weather conditions. Air samples were collected by using personal sampling pumps with sorbent tubes (OVS-2 tube: 13 mm quartz filter; XAD-2 140/270 mg). The personal sampling pump was set and calibrated with a flow rate of 1 L/min. The sorbent tube was placed in the subjects' breathing zone and the personal pump was worn around the waist. The personal pump evacuates the air through the solid sorbent tube. Personal air samples were collected while vegetable growers mixed and loaded the pesticides outdoors and while walking and spraying the pesticides in the farm area. The average time of sample collection was 27.6 minutes or 0.46 hours (range 10.2–54 minutes or 0.17–0.9 hours). After the growers finished spraying, the exposure time of vegetable growers was recorded from the timer on the personal pump. The vegetable growers only sprayed pesticides once a day, usually during the morning hours from 5:00 AM–8:00 AM when the winds are calm. Two to three samples were collected per day. Pesticides are not sprayed when it is raining so samples were not collected on rainy days. The personnel pump was calibrated using a rotameter in the lab everyday before sample collection. The sampling method for measuring the pesticide air concentration followed the NIOSH manual of analytical methods, number 5600 [5, 6]. 

The plastic cap and polytetrafluoroethylene (PTPE) retainer ring of the sorbent tube was removed for analyses. The quartz filter and front XAD-2 section was transferred to a 4 mL vial and the short polyurethane foam plug along with back-up XAD-2 section was transferred to a separate 4-mL vial. Desorbing solvent (2 mL of acetone/toluene solution: 1/9) was added to each vial and let to stand for 30 minutes. The sample was then extracted in an ultrasonic bath for 30 minutes and concentrated by evaporation with nitrogen gas and reconstituted with 500 *μ*L of desorbing solvent for gas chromatography with flame photo detector (GC/FPD) analysis. An Agilent 6890 GC/FPD was used for quantification of OPP compounds. The retention times of dicrotofos, chlorpyrifos, and profenofos were 13.924, 16.917, and 20.255 minutes, respectively.

After collecting air samples from the vegetable growers, air samples were collected from the reference subjects during latex collection from the rubber plants. Sampling conditions and collection methods for the reference subjects were the same as for the vegetable growers. The sampling duration determined for the vegetable growers was also used for nonapplicators, 27.6 minutes.

### 2.2. Quality Control

A calibration curve using the external mixed standards of chlorpyrifos, dicrotophos, and profenofos was created for each compound to be quantified at concentrations of 1, 2, 4, 8, and 10 *μ*g/mL. Calibration standards were run before samples were injected and all measurements were performed in the ranges of linearity found for each compound. The limit of method detections (MDLs) for chlorpyrifos, dicrotophos, and profenofos were 0.0016, 0.005, and 0.003 mg/m^3^, respectively. The validation data showed quantitative recoveries at 2 ppm of the three standards mixed (chlorpyrifos, dicrotophos, and profenofos). Percent recoveries of spiked samples with chlorpyrifos, dicrotophos, and profenofos in OVS-2 air sample tubes were in the range of 96–110, 102–104, and 96–106%, respectively.

### 2.3. Air Inhalation Exposure Assessment

Air inhalation exposure was calculated by the following algorithm [7]:

(1) $$\text{ADD}=\frac{C_{s}\times \text{IR}}{\text{BW}},$$

where ADD is Average Daily Dose for air inhalation (mg/kg/day), *C*_*s*_ is concentration of pesticides in air (mg/m^3^), IR is inhalation rate (m^3^/day), (for vegetable growers average breathing rate = 1.472 m^3^/day, calculated from breathing rate in heavy activity (3.2 m^3^/hr)^8^ and multiply by 0.46 hr/day for average spraying pesticide period of vegetable farmers. For reference group breathing rate and breathing time use as same as vegetable growers = 1.472 m^3^/day) BW = body weight (kg) (65 kg; average body weight of vegetable growers).

### 2.4. Risk Characterization

Based on toxicology criteria and potential for exposure, the Health Effects Division (HED) of the US Environmental Protection Agency has conducted inhalation exposure assessments for occupational handlers. Noncancer inhalation health risk estimates are expressed in terms of the Margin of Exposure (MOE). The margin of exposure (MOE) is used to convey Noncancer health risks. The MOE is the relationship of the pesticides no adverse observable adverse effects level (NOAEL), or the lowest adverse observable effects level (LOAEL), to the pesticides approximate level of exposure. The MOE is compared to the pesticides uncertainty factor; an MOE greater than the uncertainty factor reflects a possible health concern [8]. 

The NOAELs of chlorpyrifos for inhalation in acute effect was 0.1 mg/kg·day [9]. Dicrotophos LOAELs for inhalation acute effects was 0.5 mg/kg·day [10]. Profenofos LOAELs for inhalation acute effects was 9.7 mg/kg/day [11]

(2) $$\text{MOEs}=\frac{\text{NOAEL}\left(\text{mg}/\text{kg}\cdot \text{day}\right)}{\text{ADDs}\left(\text{mg}/\text{kg}\cdot \text{day}\right)}.$$

LOAEL can be substituted for NOAEL in (2), but then it is desirable to have a larger MOE.

### 2.5. Statistical Analyses

This is a cross-sectional study design. Data was analyzed using SPSS 16.0. Descriptive statistics were used to describe and summarize the data. Tests of normality were also checked. Since the air samples produced a skewed distribution, the Mann Whitney-U test was used to compare the two study groups (vegetable growers versus reference) and seasons (dry versus wet). 

## 3. Results

The vegetable grower and reference groups had similar distributions of gender, age, and weight. The surveys showed that 32 males and 1 female made up the vegetable growers with an average age of 39 years and an average weight of 65 kg. The reference group, or control group, consisted of 16 males and 1 female. The average age of the reference group was 40 years, and the average weight of the reference group was 65 kg. The vegetable growers average spray time was 0.46 hrs/day (ranging 0.17–0.9 hrs/day). 

**Figure 2:**
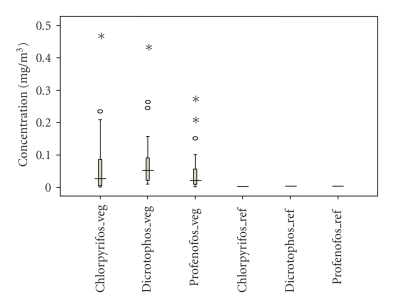
Concentrations of organophosphate pesticide exposure to vegetable growers (*n* = 33) and the reference group (*n* = 17) in Bang-Rieng (veg: vegetable growers; ref: reference group). The boxplots represent the minimum, 25th percentile, median, 75th percentile, and maximum concentrations. The open circles and stars represent mild outliers and extreme outliers, respectively.

**Figure 3:**
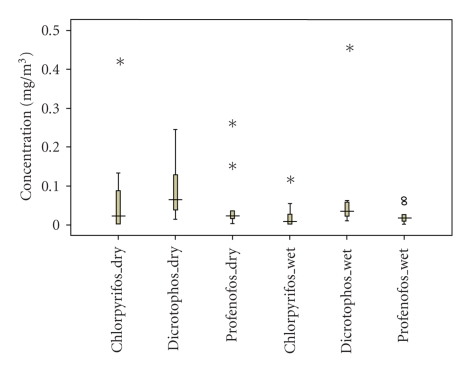
Concentration of organophosphate pesticide expose to Bang-Rieng vegetable growers during the dry (*n* = 33) and wet seasons (*n* = 33). The boxplots represent the minimum, 25th percentile, median, 75th percentile, and maximum concentrations. The open circles and stars represent mild outliers and extreme outliers, respectively.

Chlorpyrifos, dicrotophos, and profenofos were found in all the vegetable grower's air samples. Figure 2 shows the medians and distributions of all three pesticides. Results showed that median concentrations of OPPs in farm area air samples were in the range of 0.022–0.056 mg/m^3^. All reference area samples were below the analytic limit of detection, for all three sampled pesticides. The concentrations of chlorpyrifos, dicrotophos, and profenofos were significantly higher in the farm areas compared to the reference areas, during both the wet and dry seasons (Mann Whitney-U (MWU) Test; MWU, *P* ≤ .05). Figure 3 shows in the farm areas, there was no difference in chlorpyrifos, dicrotophos and profenofos concentrations between the dry and the wet seasons (MWU, *P* = .197,. 469, and .160, resp.).

The chlorpyrifos concentrations of all samples in short-term exposure during both the wet and dry seasons did not exceed Recommended Exposure Limit (REL) of chlorpyrifos = 0.6 mg/m^3^ as recommended by the American Conference of Governmental Industrial Hygienist (ACGIH) [12]. The profenofos concentrations of all samples in short-term exposure during both the wet and dry seasons did not exceed.* Occupational Exposure Limit (OEL) of profenofos = 3 mg/* *m* ^3^ *as suggestion by Syngenta Crop Protection, Inc *[13]. But the 3% of all samples was found dicrotophos concentrations in short-term exposure during both the wet and dry exceed Permission exposure limits (PELs) = 0.25 mg/m^3^ as recommended by the National Institute for Occupational Safety and Health [14]. 

### 3.1. Dose and Risk Characterization

The Margin of Exposure (MOE) is a ratio of the No Observed Adverse Effects Level (NOAEL) of chlorpyrifos, the Lowest Observed Adverse Effects Level (LOAEL) of dicrotophos, and profenofos to exposure dose. For chlorpyrifos, the US EPA has set an MOE of 100 as the US EPA MOE approach that has shown to be adequate protection for workers (US EPA, 1999) [9]. The MOE for dicrotophos is 1000 and 300 for profenofos (US EPA, 1999) [10, 11]. 

The estimated inhalation dose for chlorpyrifos, dicrotophos, and profenofos are presented in Table 1. To compare dose with effect level was presented as MOE in Table 2. Table 2 shows that 39% of vegetable grower's air samples had chlorpyrifos levels below the US EPA MOE approach and 87% of the samples had dicrotophos levels below the US EPA MOE approach. None of MOE of profenophos was below the US EPA MOE approach. The MOEs of chlorpyrifos, dicrotophos, and profenofos are not a concern in the reference area since measurable levels were not detected in these areas. 

## 4. Discussion and Conclusions

This is a study of 33 vegetable farmers in Bang-Rieng, Thailand. Vegetable growers do not wear proper protective. It was found that 39% and 87% of vegetable growers are at risk of inhalation exposures to chlorpyrifos and dicrotophos organophosphate insecticides that exceeded EPA recommended, respectively. Whereas no vegetable growers had risk concern inhalation exposure to profenofos organophosphate insecticides that exceeded EPA recommended.

The US investigated agricultural (various PPE or engineering controls) inhalation exposure to chlorpyrifos and dicrotophos. It was found that for 16% (9 of 56 scenarios) of chlorpyrofos exposure [9] and 8% (2 of 24 scenarios) of dicrotophos exposure exceeded EPA recommendations [10]. No agricultural worker (individual wearing long pants, a long-sleeve shirt, shoes and socks, no gloves. and no respirator) had inhalation exposure to profenofos organophosphate insecticides that exceeded EPA recommendations [11].

The result found that a higher percent of agricultural workers in Thailand were exposed to organophosphate pesticides (including chlorpyrifos and dicrotophos) than US agricultural workers. This is due to the fact that US agricultural workers wear the appropriate PPE and have better engineering control. The vegetable growers should be required to wear protective masks when working with pesticides and educated on the proper use of pesticides. Administrative changes such as rotating workers and scheduling application times differently may help lower the risk of exposure as well.

Data analysis also showed that the vegetable growers were exposed to a higher level of organophosphate (0.022–0.056 mg/m^3^) than the reference group (<0.0016–<0.005 mg/m^3^). Three percent of the samples showed that short-term exposure to dicrotophos during both the wet and dry seasons exceeded the permissible exposure limits (PELs) of 0.25 mg/m^3^ as recommended by the National Institute for Occupational Safety and Health. Whereas chlorpyrifos and profenofos did not exceed the short time exposure Recommended Exposure Limit (REL) or the Occupational Exposure Limit (OEL).

Comparisons of pesticide exposure to previous studies in Thailand found that the chlorpyrifos exposure of 33 vegetable growers (0.0016–0.4537 mg/m^3^) in the vegetable farm area was slightly less than the chlorpyrifos exposure of 31 rice farmers in the rice fields (ranging from 0.0216–0.5500 mg/m^3^). This may be a result of the amount of pesticides used on vegetable farms (vegetable growers farm area: 0.8 hectare) being less than rice farms (rice farmer area: 1.6–3.2 hectare) [15].

The comparison of pesticide concentrations with ACGIH/NIOSH guidelines and the MOE, analysis found that some of the dicrotophos samples (chlorpyrifos: 3% exceeded REL and 87% exceeded MOE) exceeded the fixed values. It was also found that profenofos (profenofos: 0% exceeded OEL and 0% exceeded MOE) did not exceed the fixed values. The incident rates of chlorpyrifos showed that 39% of the samples exceeded the MOE. The incident rates of the MOE may be more than PEL, REL or OEL because the EPA derived a margin of exposure (MOE) from the dermal no-observed-adverse-effect level (NOAEL; mg/kg/day).

The NOAEL relied on animal studies and human studies (if applicable). The uncertainty factor (UF) accounts for the uncertainty involved in extrapolating animal data for human health. The uncertain factor for occupational exposure, MOE(chlorpyrifos) >100 (i.e., 10× interspecies and 10× intraspecies variability, do not exceed HED's level concern) and MOE >300 (profenofos) or 1000 (dicrotophos) (i.e., 10× interspecies and 10× intraspecies variability, do not exceed HED's level concern the addition 3-to 10-fold cushion between NOAEL and LOAEL) [9]. The uncertainty factors derived from combination with actual data and surrogate data (for professional judgment). This is one sources of uncertainty that may not represent the exposure scenario being analyzed. However, EPA set uncertainty factors as high potential safety factors. So using the MOE and EPA to enforce standards, it may reduce pesticide exposure. 

The Occupational Safety and Health Act (OSHA) ensure a safe and healthy work environment. This is accomplished by setting occupational safety and health standards and by providing research, information, and training in the field of occupational safety and health [16]. The ACGIH limits are based on the risk of cholinesterase inhibition associated with exposure to chlorpyrifos and dicrotophos [17, 18]. The workers are viewed as generally healthier than the general population and having chosen to face certain risks and be compensated for this risk through payment of wages, but neither assumption is necessarily correct.

From the reasons above the degree of pesticide exposure with ACGIH/NIOSH guidelines and the MOE analysis was different. Congress has forbidden OSHA from conducting inspection at farms that employ 10 or fewer persons, leaving EPA as the only agency with meaningful enforcement authority in many situations [19]. Each vegetable farm in Thailand consists of fewer persons. So Thailand should enforce pesticide use by the EPA MOE since the EPA sets as high potential safety factor.

Even though it is important to estimate inhalation exposure, it is not enough to estimate occupational exposure. Measuring inhalation exposure alone may underestimate each subject's true dose because of the known propensity for dermal exposure of these three agents and the high probability of skin exposure during mixing and backpack-spray application. The potential for systematic toxicity can be observed following dermal exposure. Therefore, ACGIH set short-term exposure levels (STELs) of chlorpyrifos with skin notations. The skin notation indicates that the cutaneous route of exposure (including mucous membrane and eye) contributes to the overall exposure. In addition, OSHA set a PEL for dicrotophos with skin notations as well. Even though this research did not focus on dermal exposure, it is recommended that dermal exposure be investigated in future studies. Dermal exposure is a concern in Thailand since Thai farmers do not wear appropriate personal protective equipment during the loading, mixing, and spraying of pesticides.

This study is a preliminary aggregate risk assessment (of all pesticides used on farms) of inhalation exposure among farmers in Thailand. The information obtained from this study is useful for risk management and risk communication in the Bang-Rieng agricultural community. It also provides baseline information for local and national government to make decisions relevant to farmer health. Individual health risk assessments were also reported to each vegetable grower who was also provided guidance on protection from pesticide exposure.

## Figures and Tables

**Table 1:** Estimated inhalation dose of organophosphate pesticide residues for vegetable growers.

Pesticide	Effect Level (mg/kg/d)	Dose (mg/kg/d)				
		GEM*	25%	50%	75%	90%
Chlopyrifos	0.1 (NOAEL)	0.0006	0.0003	0.0007	0.0015	0.0030
Dicrotophos	0.5 (LOAEL)	0.0013	0.0005	0.0016	0.0024	0.0056
Profenofos	9.7 (LOAEL)	0.0007	0.0003	0.0006	0.0031	0.0090

GEM* = Geometric mean.

**Table 2:** Margin of Exposure (MOE) of organophosphate pesticide residues for vegetable growers.

Pesticide	Frequency Below EPA MOE approach	Margin of exposure (MOE)				
		GEM*	25%	50%	75%	90%
Chlopyrifos	39%	167	333	143	67	33
Dicrotophos	87%	385	1000	312	208	89
Profenofos	0%	13857	32333	16167	3129	1077

US EPA MOE Chlorpyrifos =100, dicrotophos =1000, profenofos =300

MOE < US EPA MOE approach mean risk concern.
